# Do PPAR*γ* Ligands Suppress the Growth of Cholangiocarcinoma or the Cholangiohepatitis Induced by the Tumor?

**DOI:** 10.1155/2008/587401

**Published:** 2008-07-06

**Authors:** Satoru Suzuki, Kiyoshi Hashizume

**Affiliations:** Department of Aging Medicine and Geriatrics, Institute on Aging and Adaptation, Shinshu University Graduate School of Medicine, 3-1-1 Asahi, Matsumoto 390-8621, Japan

## Abstract

Cholangiocarcinoma is a predominantly fatal cancer, which can be difficult to treat. It has been reported that the administration of pioglitazone temporarily improved not only diabetic control, but also bile duct carcinoma-induced cholangiohepatitis. Pioglitazone is considered to have both direct and indirect mechanisms of action on the tumor-related hepatitis. Several molecules induced by thiazolidinedione, including Smad pathway-related molecules, adipokines, and other lipid metabolism-related proteins, may directly or indirectly suppress tumor development and/or tumor-induced cholangiohepatitis. Although the most frequent and critical side effect of thiazolidinedione is drug-induced hepatitis, it can probably be avoided by careful monitoring of serum hepatic enzyme levels. Thiazolidinedione should be considered for management of tumor-induced hepatitis in the presence of diabetes unless severe side effects occur.

## 1. INTRODUCTION

The primary effects of thiazolidinedione, a peroxisome proliferator-activated receptor *γ* (PPAR*γ*) agonist, are the reduction of insulin resistance and improvement of insulin sensitivity, resulting in reduction of fasting plasma glucose, insulin, and free fatty acid levels [1].

Cholangiocarcinoma is a predominantly fatal cancer, which can be difficult to treat. We reported previously that administration of the thiazolidinedione, pioglitazone, in a 73-year-old male patient who developed cholangiocarcinoma with cholangiohepatitis and diabetes improved not only diabetic control, but also the tumor-induced cholangiohepatitis, and improved the patient's quality of life [2]. One and half years after treatment, the patient again showed deterioration of cholangiohepatitis and diabetic control. He finally died of obstructive jaundice.

There are two possible mechanisms to explain the initial improvement of hepatitis in our case: the PPAR*γ* ligand may have directly suppressed the abnormal cell growth, or the PPAR*γ* ligand may have indirectly suppressed tumor growth after the ligand improved hepatitis and/or diabetes.

In this review, we discuss the mechanisms of the temporary beneficial effects of the agent, especially the above two possibilities, with regard to the literature concerning PPAR*γ* and cholangiohepatitis. In addition, we also discuss the positive choice of thiazolidinedione, despite elevated serum concentrations of hepatic enzymes.

## 2. DIRECT EFFECTS ON THE DEVELOPMENT OF CHOLANGIOCARCINOMA

These mechanisms were supported by the results of basic experiments using various cholangiocarcinoma cell lines [3–6]. PPAR*γ* ligand mediates the inhibition of cholangiocarcinoma cell growth through p53-dependent mechanisms [3]. The PPAR*γ* ligand, 15-deoxy-delta 12,14-PGJ2, induces apoptosis in cholangiocarcinoma cell lines although regulation of apoptosis-related protein expression varies [4, 5], while artificial regulation of PPAR*γ* expression in cholangiocarcinoma cell lines suggests that PPAR*γ* may actually promote tumor cell growth viathe Smad pathway [6]. It has been reported that PPAR*γ* ligands can suppress proliferation and induce apoptosis although PPAR*γ* itself may have divergent effects on cellular growth in cholangiocarcinoma cell lines [7].

## 3. INDIRECT EFFECTS ON THE DEVELOPMENT OF CHOLANGIOCARCINOMA

Thiazolidinedione seems not to improve insulin sensitivity and glucose disposal by direct effects on either the liver or muscle. PPAR*γ* is expressed preferentially in adipose tissue, and its levels of expression in the liver and skeletal muscle are low [8]. Thus, it is more likely that the primary effects of these drugs are on adipose tissue, followed by secondary benefits on other target tissues of insulin [9]. In our case, there was no evidence that pioglitazone directly reduced the tumor size. In contrast, cholangiohepatitis was improved by administration of this agent. In addition, the progressive cholangiohepatitis was probably related to the cholangiocarcinoma. In general, cholangiocarcinoma development is based possibly upon the cytotoxicity of bile constituents, that is, cytotoxic bile acids and lysolecithins. These humoral factors may affect tumor progression. Thus, it was suggested that pioglitazone indirectly improves cancer-mediated inflammation, such as cholangiohepatitis, rather than directly suppressing tumor growth.

**Figure 1:**
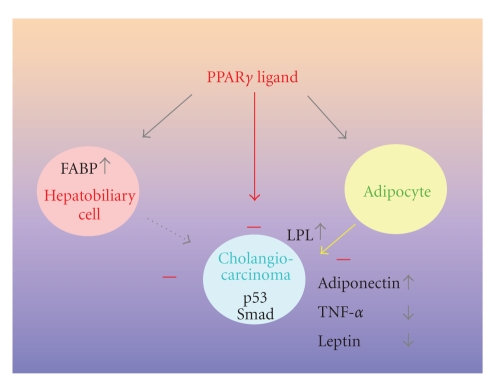
Inhibitory effects of PPAR*γ* ligand on the development of cholangiocarcinoma. PPAR*γ* ligand directly suppresses tumor progression through p53 and Smad pathways (red arrow) and also stimulates adipocyte and hepatobiliary cells (gray arrow). Secretion of adipokines (TNF-*α*, adiponectin, and leptin) and production of lipid-related proteins (FABP and LPL) are regulated by PPAR*γ* ligand. Up- and downregulation of various gene signals from adipocytes (yellow line) and hepatobiliary cells (broken line) promoted suppression of tumor growth. As a result, PPAR*γ* indirectly suppressed tumor growth of cholangiocarcinoma through adipocytes and hepatobiliary cells. Currently, evidence of suppressive signals from hepatobiliary cells to cholangiocarcinoma is unavailable (broken line). FABP: fatty acid binding protein, LPL: lipoprotein lipase, TNF-*α*: tumor necrosis factor-*α*.

As mentioned above, it is now generally accepted that adipose cells send molecular signals, including cytokines, to other tissues. Thus, it is possible that PPAR*γ* activation controls one or more genes that regulate systemic tumor promotion (see Figure 1). The interesting candidate genes in this regard are TNF-*α*, adiponectin, and leptin. Other lipid-related genes regulated by PPAR*γ* ligands, such as lipoprotein lipase and fatty acid binding protein, may also control tumor development [10].

### 3.1. TNF-*α*

Thiazolidinedione reduces TNF-*α* expression in human and rodent adipocytes [11]. A series of studies using cholangiocarcinoma cell lines demonstrated that TNF-*α* itself attenuates the growth of cholangiocarcinoma cells and induces apoptosis [11–13]. However, several recent studies have demonstrated that TNF-*α* promotes invasiveness and accelerates migration of cholangiocarcinoma cells [14–16]. These observations imply that the suppression of TNF-*α* production may attenuate the progressive invasion of tumor cells into healthy hepatobiliary cells.

### 3.2. Adiponectin and leptin

PPAR*γ* agonists have been reported to increase the expression and circulating levels of adiponectin, an adipocyte-derived protein with insulin-sensitizing activity [17], in diabetic rodents and in patients with type 2 diabetes [18]. There have been many reports, especially in breast cancer, that adiponectin plays roles in the inhibition of tumor cell growth [19]. The expression of leptin, a suppressor of feeding behavior, is negatively regulated by thiazolidinediones [20]. Leptin has been reported to induce tumor development in breast cancer [21], suggesting that suppression of leptin secretion may reduce tumor progression.

### 3.3. Other lipid-related proteins

Other lipid-related proteins, such as lipoprotein lipase (LPL) and fatty acid binding proteins (FABPs), are positively regulated by the PPAR*γ* ligand GW1929 [22]. Although there have been no studies related to cholangiocarcinoma and these lipid-associated proteins, there is a great deal of evidence that the proteins promote reduction of tumor growth. Intestinal polyp formation was suppressed by increasing LPL activity [23]. As FABPs play roles not only as lipid chaperones but also as free radical scavengers, the molecules may affect tumor progression through the oxidative stress pathways. The protein expression of liver FABP was reduced in neoplastic lesions of CuZn superoxide dismutase-deficient mice [24]. It has been reported that FABP reduces cellular damage from hypoxia/reoxygeneration [25]. These lipid-related proteins may also play roles in the reduction of tumor growth and/or suppression of tumor-mediated liver damage.

## 4. HEPATIC SIDE EFFECTS

The most frequent and critical side effect of thiazolidinedione that must be taken into consideration before starting thiazolidinedione administration in cases of cholangiocarcinoma is drug-induced hepatitis. Although some data are available from animal studies suggesting that hepatic toxicity may be a characteristic of the thiazolidinedione class [26], current clinical evidence indicates that pioglitazone treatment does not result in liver toxicity [27]. However, this agent causes mild transient increases in serum ALT levels. The FDA recommends monitoring ALT levels and not using these drugs in patients with liver disease [28]. Moreover, it was reported that patients receiving pioglitazone may develop serious liver injury and should be monitored for evidence of hepatitis [29].

Unlike other existing antidiabetic medications that show a very rapid onset of activity, pioglitazone and rosiglitazone exhibit a characteristic delay of 4–12 weeks in the onset of their therapeutic effects. It has been suggested that thiazolidinedione should be continued for at least one month to obtain results. In our case, initial improvement of the elevated hepatic enzymes was observed two weeks after starting administration of this agent. These data indicate that the effectiveness in cases of tumor-related hepatitis could be assessed within two weeks rather than 4–12 weeks when diabetic control is obtained.

## 5. PERSPECTIVES

Taken together with these considerations, as PPAR*γ* ligands are probably effective in the suppression of tumor development, especially on the reduction of tumor invasiveness through molecular signals from adipocytes, thiazolidinedione should be chosen not only for diabetic control, but also as an attenuator of tumor progression in patients with diabetes. Drug-induced hepatitis can be avoided by meticulous monitoring of serum hepatic enzyme levels.

## References

[1] Mudaliar S, Henry RR (2001). New oral therapies for type 2 diabetes mellitus: the glitazones or insulin sensitizers. *Annual Review of Medicine*.

[2] Suzuki S, Mori J, Yamazaki M, Sato A, Hosoda W, Hashizume K (2007). Beneficial effects of pioglitazone on cholangiohepatitis induced by bile duct carcinoma. *Internal Medicine*.

[3] Han C, Demetris AJ, Michalopoulos GK, Zhan Q, Shelhamer JH, Wu T (2003). PPAR*γ* ligands inhibit cholangiocarcinoma cell growth through p53-dependent GADD45 and p21^WAF1/Cip1^ pathway. *Hepatology*.

[4] Okano H, Shiraki K, Inoue H (2003). The PPARgamma ligand, 15-deoxy-delta 12,14-PGJ2, regulates apoptosis-related protein expression in cholangio cell carcinoma cells. *International Journal of Molecular Medicine*.

[5] Kobuke T, Tazuma S, Hyogo H, Chayama K (2006). A ligand for peroxisome proliferator-activated receptor *γ* inhibits human cholangiocarcinoma cell growth: potential molecular targeting strategy for cholangioma. *Digestive Diseases and Sciences*.

[6] Han C, Demetrist AJ, Liu Y, Shelhamer JH, Wu T (2004). Transforming growth factor-*β* (TGF-*β*) activates cytosolic phospholipase A_2_
*α* (cPLA_2_
*α*)-mediated prostaglandin E_2_(PGE)_2_/EP_1_ and peroxisome proliferator-activated receptor-*γ* (PPAR-*γ*)/Smad signaling pathways in human liver cancer cells: a novel mechanism for subversion of TGF-*β*-induced mitoinhibition. *The Journal of Biological Chemistry*.

[7] Shimizu Y, Demetris AJ, Gollin SM (1992). Two new human cholangiocarcinoma cell lines and their cytogenetics and responses to growth factors, hormones, cytokines or immunologic effector cells. *International Journal of Cancer*.

[8] Auboeuf D, Rieusset J, Fajas L (1997). Tissue distribution and quantification of the expression of mRNAs of peroxisome proliferator-activated receptors and liver X receptor-alpha in humans: no alteration in adipose tissue of obese and NIDDM patients. *Diabetes*.

[9] Combs TP, Wagner JA, Berger J (2002). Induction of adipocyte complement-related protein of 30 kilodaltons by PPAR*γ* agonists: a potential mechanism of insulin sensitization. *Endocrinology*.

[10] Spiegelman BM (1998). PPAR-*γ*: adipogenic regulator and thiazolidinedione receptor. *Diabetes*.

[11] Arner P (2003). The adipocyte in insulin resistance: key molecules and the impact of the thiazolidinediones. *Trends in Endocrinology and Metabolism*.

[12] Utaisincharoen P, Ubol S, Tangthawornchaikul N, Chaisuriya P, Sirisinha S (1999). Binding of tumour necrosis factor-alpha (TNF-*α*) to TNF-RI induces caspase(s)-dependent apoptosis in human cholangiocarcinoma cell lines. *Clinical & Experimental Immunology*.

[13] Nzeako UC, Guicciardi ME, Yoon J-H, Bronk SF, Gores GJ (2002). COX-2 inhibits Fas-mediated apoptosis in cholangiocarcinoma cells. *Hepatology*.

[14] Ohira S, Sasaki M, Harada K (2006). Possible regulation of migration of intrahepatic cholangiocarcinoma cells by interaction of CXCR4 expressed in carcinoma cells with tumor necrosis factor-*α* and stromal-derived factor-1 released in stroma. *The American Journal of Pathology*.

[15] Ishimura N, Isomoto H, Bronk SF, Gores GJ (2006). Trail induces cell migration and invasion in apoptosis-resistant cholangiocarcinoma cells. *American Journal of Physiology*.

[16] Tanimura Y, Kokuryo T, Tsunoda N (2005). Tumor necrosis factor *α* promotes invasiveness of cholangiocarcinoma cells via its receptor, TNFR2. *Cancer Letters*.

[17] Yamauchi T, Kamon J, Waki H (2001). The fat-derived hormone adiponectin reverses insulin resistance associated with both lipoatrophy and obesity. *Nature Medicine*.

[18] Yang W-S, Jeng C-Y, Wu T-J (2002). Synthetic peroxisome proliferator-activated receptor-*γ* agonist, rosiglitazone, increases plasma levels of adiponectin in type 2 diabetic patients. *Diabetes Care*.

[19] Rose DP, Haffner SM, Baillargeon J (2007). Adiposity, the metabolic syndrome, and breast cancer in African-American and white American women. *Endocrine Reviews*.

[20] De Vos P, Lefebvre A-M, Miller SG (1996). Thiazolidinediones repress *ob* gene expression in rodents via activation of peroxisome proliferator-activated receptor *γ*. *The Journal of Clinical Investigation*.

[21] Vona-Davis L, Rose DP (2007). Adipokines as endocrine, paracrine, and autocrine factors in breast cancer risk and progression. *Endocrine-Related Cancer*.

[22] Way JM, Harrington WW, Brown KK (2001). Comprehensive messenger ribonucleic acid profiling reveals that peroxisome proliferator-activated receptor *γ* activation has coordinate effects on gene expression in multiple insulin-sensitive tissues. *Endocrinology*.

[23] Mutoh M, Niho N, Wakabayashi K (2006). Concomitant suppression of hyperlipidemia and intestinal polyp formation by increasing lipoprotein lipase activity in *Apc*-deficient mice. *Biological Chemistry*.

[24] Elchuri S, Naeemuddin M, Sharpe O, Robinson WH, Huang T-T (2007). Identification of biomarkers associated with the development of hepatocellular carcinoma in CuZn superoxide dismutase deficient mice. *Proteomics*.

[25] Wang G, Gong Y, Anderson J (2005). Antioxidative function of L-FABP in L-FABP stably transfected Chang liver cells. *Hepatology*.

[26] Boelsterli UA, Bedoucha M (2002). Toxicological consequences of altered peroxisome proliferator-activated receptor *γ* (PPAR*γ*) expression in the liver: insights from models of obesity and type 2 diabetes. *Biochemical Pharmacology*.

[27] Chilcott J, Tappenden P, Jones ML, Wight JP (2001). A systematic review of the clinical effectiveness of pioglitazone in the treatment of type 2 diabetes mellitus. *Clinical Therapeutics*.

[28] Tolman KG, Fonseca V, Tan MH, Dalpiaz A (2004). Narrative review: hepatobiliary disease in type 2 diabetes mellitus. *Annals of Internal Medicine*.

[29] May LD, Lefkowitch JH, Kram MT, Rubin DE (2002). Mixed hepatocellular-cholestatic liver injury after pioglitazone therapy. *Annals of Internal Medicine*.

